# Overexpression of *OsHSP18.0-CI* Enhances Resistance to Bacterial Leaf Streak in Rice

**DOI:** 10.1186/s12284-017-0153-6

**Published:** 2017-04-17

**Authors:** Yanhu Ju, Hongjuan Tian, Ruihua Zhang, Liping Zuo, Guixiu Jin, Qian Xu, Xinhua Ding, Xiangkui Li, Zhaohui Chu

**Affiliations:** 1 State Key Laboratory of Crop Biology, Shandong Provincial Key Laboratory of Agricultural Microbiology, Shandong Agricultural University, Tai an, 271018 Shandong People’s Republic of China; 2 Rice Research Institute, Linyi Academy of Agricultural Science, Linyi, 276012 Shandong People’s Republic of China; 3 Present address: Haoyunjiao Resort Party Committee Government Office, Rongcheng, Shandong People’s Republic of China; 4 State Key Laboratory of Crop Biology, College of Agronomy, Shandong Agricultural University, Tai’an, 271018 China; 5 Shandong Provincial Key Laboratory of Agricultural Microbiology, College of Plant Protection, Shandong Agricultural University, Tai’an, 271018 China

**Keywords:** Bacterial leaf streak, Disease resistance, Heat shock protein, *OsHsp18.0-CI*, *Xanthomonas oryzae* pv. *oryzicola*

## Abstract

**Background:**

The small heat shock proteins represent a large family of proteins that respond to a wide range of abiotic and biotic stresses. OsHsp18.0-CI confers tolerance to salt and cadmium and interacts with viral RNA-dependent RNA polymerase (RdRp). However, the direct function of OsHsp18.0-CI in resistance against biotic stresses remains unclear in rice.

**Results:**

Here, we report that the expression of *OsHsp18.0-CI* was up-regulated upon inoculation with RS105, a strain of *Xanthomonas oryzae* pv. *oryzicola* (*Xoc*) that causes bacterial leaf streak in rice. In comparison with wild-type, *OsHsp18.0-CI* overexpression (OE) lines exhibited enhanced resistance to RS105, whereas repression lines exhibited compromised resistance to RS105. In addition, the transcriptional profiles of wild type and OE lines were compared with and without inoculation with RS105. After inoculation with RS105, most of the genes with up-regulated expression were commonly stimulated in the wild type and OE lines, with stronger induction in the OE lines than in wild type.

**Conclusion:**

Our study reveals that *OsHsp18.0-CI* positively regulates resistance to *Xoc* by mediating an enhanced version of the basal defense response in rice.

**Electronic supplementary material:**

The online version of this article (doi:10.1186/s12284-017-0153-6) contains supplementary material, which is available to authorized users.

## Background

Bacterial leaf streak (BLS) caused by the gram-negative bacterial pathogen *Xanthomonas oryzae* pv. *oryzicola* (*Xoc*) is a globally important disease affecting rice production. This pathogen often penetrates the leaf through stomata or wounds and propagates in the apoplast of mesophyll tissue, finally resulting in water-soaked lesion symptoms (NIÑO‐Liu et al. 2006). *Xoc* normally causes yield losses of approximately 20% depending on the rice variety and climatic conditions (Ou 1985). Since first reported in southern China in 1957, *Xoc* has spread to most areas of Hainan province, southern China, southwest China and central China, becoming one of the most devastating quarantine rice diseases (NIÑO‐Liu et al. 2006; Xu et al. 2008).

Breeding disease-resistant varieties is an ideal strategy to manage BLS disease. However, BLS resistance has been considered quantitatively inherited in rice. More than 13 quantitative trait loci (QTLs) have been mapped from the *indica* rice varieties Acc8558 and Dular (Tang et al. 2000; Chen et al. 2006). The major QTL is *qBlsr5a*, in the short arm of chromosome 5, which has the largest effect and explains approximately 14% of phenotypic variation (Tang et al. 2000; Han et al. 2008). It was identified that *qBlsr5a* is mainly controlled by *xa5*, a major gene for resistance to bacterial blight caused by *Xanthomonas oryzae* pv. *oryzae* (Xie et al. 2014). Recently, a new dominant resistance locus named *Xo1* has been identified in the qualitative resistance against the African clade of *Xoc* strains of the American heirloom rice variety Carolina Gold Select (Triplett et al. 2016). Interestingly, this resistance can be triggered by a transcription activator-like (TAL) effector in *Xoc* (Triplett et al. 2016). Alternatively, a non-host resistance gene, *Rxo1*, which encodes a typical plant resistance protein containing a nucleotide-binding site-leucine rich repeat (NBS-LRR) from maize, presents qualitative resistance to BLS in rice (Zhao et al. 2005). In addition to genetic mapping, some defense-related (*DR*) genes have been reported to exhibit up-regulated expression upon *Xoc* inoculation to positively or negatively regulate the BLS resistance (Zhou et al. 2010; Kou and Wang 2010; Xu et al. 2013). Overexpression of the NBS-LRR-type gene *DEPG1* increased susceptibility to *Xoc* strain RS105, implying negative regulation of rice immunity (Guo et al. 2012). Consistent with negative regulation of rice immunity, suppression of the expression of *DR* genes also enhances the resistance to BLS, such as *OsWRKY45-1* (Tao et al. 2009), *OsMPK6* (Shen et al. 2010) and *NRRB*, which encodes a receptor-like cytoplasmic kinase (Guo et al. 2014). By contrast, positive regulation mechanisms of resistance to *Xoc* have been poorly explored in rice. Overexpression of *OsPGIP4*, which encodes a polygalacturonase-inhibiting protein, and *GH3-2* which encodes an indole-3-acetic acid-amido synthetase, significantly enhance resistance to BLS in rice (Fu et al. 2011; Feng et al. 2016).

Heat shock proteins (HSPs) are found across a wide diversity of organisms. HSPs are chaperones that assist in protein folding and prevent irreversible protein aggregation (Waters 2013). They include a number of conserved protein families: HSP100s, HSP90s, HSP70s, HSP60s, and HSP20s or small HSPs (sHSPs). Among HSPs, the sHSP family is one of the most abundant and complex groups, and the monomers of these proteins range in size from 12 kDa to 42 kDa. As this alternative name of the HSP20 family suggests, most sHSPs are in the range of 15 kDa to 22 kDa, and these proteins contain a highly conserved central domain called the Alpha Crystallin Domain (ACD), which is involved in dimerization (Jaspard and Hunault 2016). There are 40 ACD-containing genes, of which 23 constitute sHSPs in rice (Sarkar et al. 2009). These sHSPs are stimulated in response to a wide range of abiotic stresses. For instance, the expression of *Os03g16030*, formerly named *Hsp18.0-CI*, *OsMSR3* and *OsSHSP1*, was elevated in response to treatment with anoxia, heat, cold, salt, drought, L-azetidine-2-carboxylic acid (AZC) and cadmium (Guan et al. 2004; Sarkar et al. 2009; Cui et al. 2013; Ham et al. 2013). Consistent with their up-regulated expression patterns, heterogeneous overexpression of *Os03g16030* in Arabidopsis enhances tolerance to salt and cadmium stresses (Cui et al. 2013; Ham et al. 2013). The expression of *Os03g16030* was also activated by biotic stresses, such as *Magnaporthe grisea* (*M. grisea*) and at least seven rice viruses (Sarkar et al. 2009; Li et al. 2015). Os03g16030 has been reported to directly interact with the N-terminus of viral RNA-dependent RNA polymerase (RdRp) to change its sub-cellular localization and distribution pattern in *Nicotiana benthamiana* (Li et al. 2015). However, the function of *Os3g16030* against rice pathogens remains unclear.

Here, we determined that the expression of *Os03g16030* (named *OsHsp18.0-CI* according to Sarkar et al. 2009) was up-regulated by inoculating with *Xoc* strain RS105. Transgenic plants overexpressing *OsHsp18.0-CI* exhibited enhanced resistance to RS105, and lines with suppressed expression exhibited increased susceptibility to RS105. Using an RNA-seq strategy, we further found that the enhanced expression of defense-related genes involved in basal defense was enriched in *OsHsp18.0-CI*-overexpressing (OE) lines compared with wild-type (WT). The expression of these genes was activated more strongly in OE than in WT plants after inoculation with RS105. Our results suggest that the sHSP *OsHsp18.0-CI* positively regulates resistance to *Xoc* in rice.

## Results

### The Expression of *OsHsp18.0-CI* is Induced by *Xoc*

**Fig. 1:**
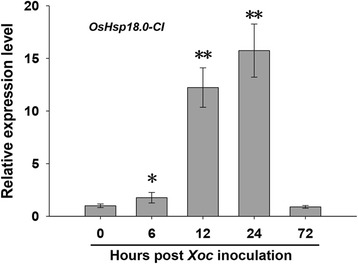
*OsHsp18.0-CI* is induced by *Xoc* inoculation. The expression level of *OsHsp18.0-CI* in response to RS105 in Shengdao806 at 0, 6, 12, 24 and 72 h post inoculation. The *bars* represent the means (three replicates for gene expression) ± SD. The significance of expression compared to 0 h at *P* values of less than 0.05 and 0.01 are marked by “*” and “**”, respectively

As the expression of *OsHsp18.0-CI* is activated by *M. grisea* and rice viruses (Sarkar et al. 2009; Li et al. 2015), we determined if it is also induced by *Xoc*. We measured the expression of *OsHsp18.0-CI* post-inoculation with the *Xoc* strain RS105 in the susceptible rice variety Shengdao 806. The transcript level of *OsHsp18.0-CI* increased quickly after 6 h post-inoculation (hpi) with RS105 (Fig. 1) and reached maximum level at 24 hpi, with an increase of 16-fold compared with the control. Expression then decreased to nearly the same level as the control at 72 hpi. This result suggests that the expression of *OsHsp18.0-CI* is induced by *Xoc*, as observed for previously reported defense-related genes (Feng et al. 2016).

### Overexpression of *OsHsp18.0-CI* Enhances Resistance Against *Xoc* in Shengdao 806

**Fig. 2:**
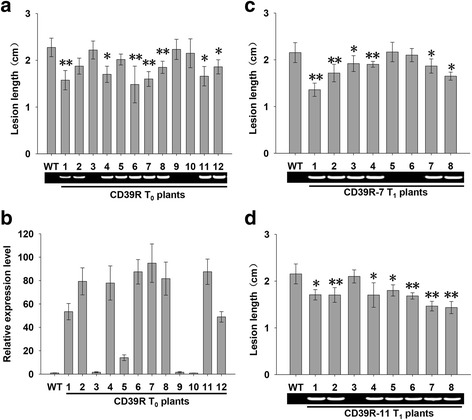
Resistance of *OsHsp18.0-CI*-overexpressing plants to the *Xoc* strain RS105. **a** Lesion lengths of the 12 individual T_0_ transgenic plants with pU1301::*OsHsp18.0-CI* (CD39R) at 14 days post inoculation (dpi). **b** Relative expression levels of *OsHsp18.0-CI* in the T_0_ transgenic plants. **c** and (**d**) Resistance of two *OsHsp18.0-CI*-overexpressing lines (CD39R-7 and CD39R-11) to *Xoc* in the T_1_ generation. The average lesion length for each plant was measured at 10 to 15 inoculation sites at 14 dpi. The gel graph indicates the plants carrying pU1301::*OsHsp18.0-CI* that were validated by PCR. The *bars* represent the means ± SD. “*” and “**” indicate significant (*t* test, *P* < 0.05) and extremely significant (*t* test, *P* < 0.01) differences in lesion lengths between wild-type and transgenic plants, respectively

To assess whether up-regulated *OsHsp18.0-CI* expression could enhance resistance against *Xoc*, *Agrobacterium*-mediated transformation of the pU1301-*OsHsp18.0-CI* construct was used to obtain 12 independent T_0_ individuals (CD39R-1 ~ CD39R-12). Nine of 12 T_0_ lines were identified as positive transgenic individuals by PCR analysis and qRT-PCR, which quantified the expression level of *OsHsp18.0-CI*. The expression level of *OsHsp18.0-CI* was significantly increased in all 9 individuals (Fig. 2b). The T_0_ individuals were inoculated with *Xoc* strain RS105 along with Shengdao806 (WT) at the seedling stage. All nine positive individuals exhibited enhanced resistance to RS105, with lesion lengths ranging from 1.58 cm to 2.02 cm, compared with an average of 2.28 cm for WT plants (Fig. 2a). The lesion length was significantly shorter in 7 of 9 OE lines (*t* test, *P* < 0.05) than in WT plants. Furthermore, the deduced lesion length in transgenic individuals was correlated (*r* =0.871, α = 0.05, *n =* 12) with the expression level of *OsHsp18.0-CI*.

To evaluate the disease resistance of transgenic lines in the T_1_ generation, two lines (CD39R-7 and CD39R-11) were selected for inoculation with RS105, with WT plants as a control. The progeny of the two T_1_ lines showed significant reductions in lesion length and were identified as positive plants that carried the construct pU1301-*OsHsp18.0-CI* (Fig. 2c and d). Two lines were further identified that exhibited resistance against RS105 in the T_2_ generation (Fig. 4a and b). Consistent with the enhanced resistance against RS105, the bacteria populations of CD39R-7 was dramatically less than control at 4 dpi and 8 dpi in leaves (Fig. 4c). These results suggested that *OsHsp18.0-CI* positively regulates resistance to *Xoc* in rice.

### Repressing *OsHsp18.0-CI* Expression Enhances Susceptibility to *Xoc*

**Fig. 3:**
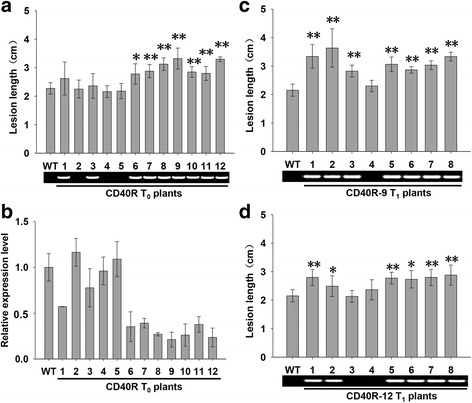
Repressing *OsHsp18.0-CI* expression enhances susceptibility to *Xoc*. **a** Lesion lengths of the 12 individual T_0_ transgenic plants with ds1301::*OsHsp18.0-CI* (CD40R). **b**Relative expression levels of *OsHsp18.0-CI* in the T_0_ transgenic plants. **c** and (**d**) The progeny of two T_1_ lines (CD40R-9 and CD40R-12) showed significant reductions in lesion length after inoculation with *Xoc*. The average lesion length for each plant was measured at 10 to 15 inoculation sites at 14 dpi. The gel graph indicates the plants carrying ds1301::*OsHsp18.0-CI* that were validated by PCR. The *bars* represent the means ± SD. “*” and “**” indicate significant (*t* test, *P* < 0.05) and extremely significant (*t* test, *P* < 0.01) differences in lesion lengths between wild-type and transgenic plants, respectively

**Fig. 4:**
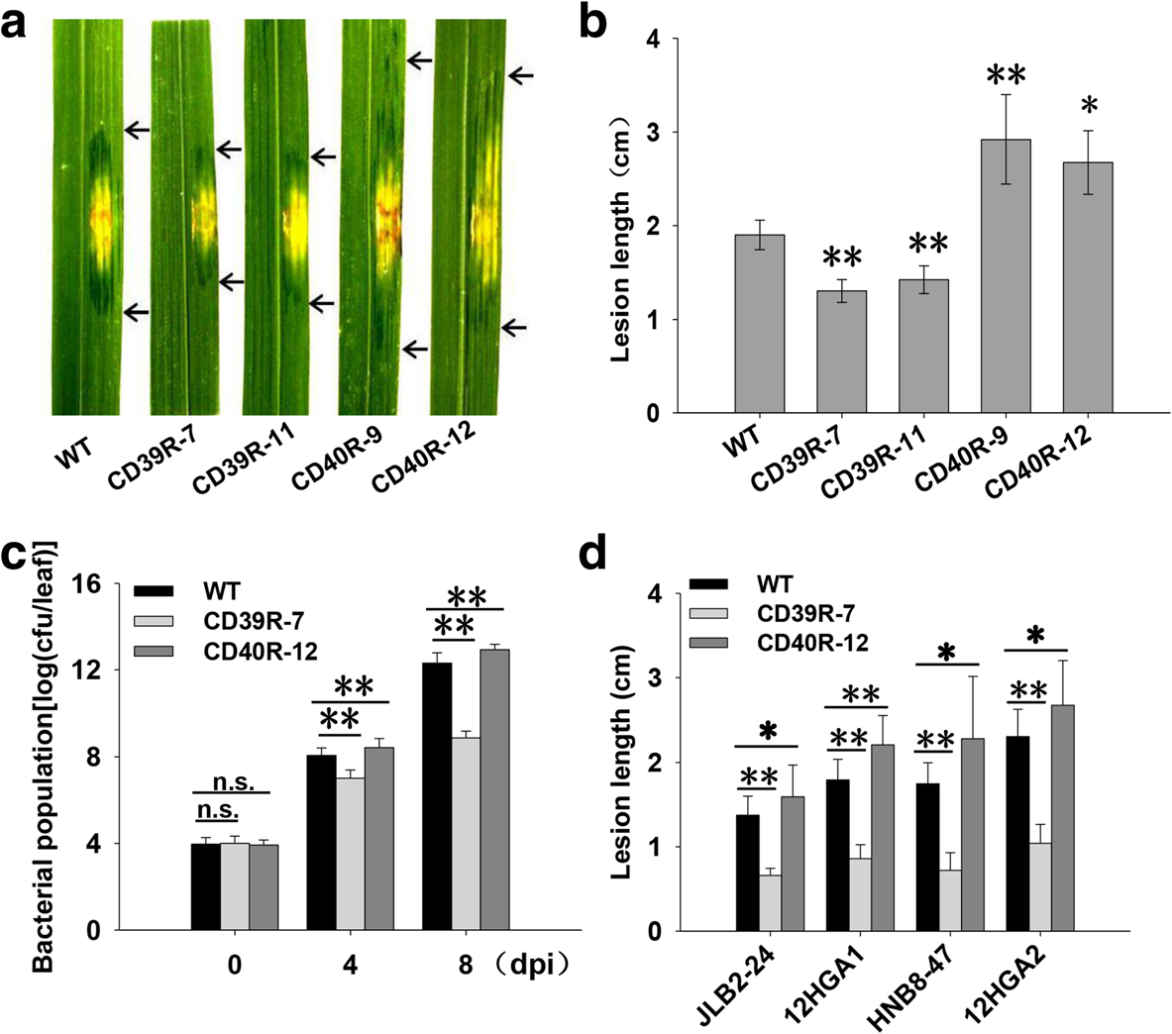
Resistance performance of transgenic plants at the T_2_ generation. **a** Representative lesion site from plants at 14 dpi with *Xoc* strain RS105. **b** Lesion length scored for the T_2_ transgenic plants of pU1301::*OsHsp18.0-CI* (CD39R-7 and CD39R-11) and ds1301::*OsHsp18.0-CI* (CD40R-9 and CD40R-12) at 14 dpi. The average lesion length for T_2_ plants was measured in 10 to 12 individuals at more than 5 inoculation sites at 14 dpi. The *bars* represent the means ± SD.**c** Bacterial population growth in leaves of CD39R-7, CD40R-12 and WT inoculated with RS105. Colonies were counted with the leaf segment up to 5 cm from the inoculation site. Error bars represent standard deviation of three independent leaves. **d** Lesion length of CD39R-7, CD40R-12 and WT inoculated with JLB2-24, HNB8-47, HGA1 and HGA2

To further identify the role of *OsHsp18.0-CI* in rice-*Xoc* interactions, we introduced the *OsHsp18.0-CI*-RNAi construct (CD40R) into Shengdao 806 to silence the expression of *OsHsp18.0-CI* by *Agrobacterium*-mediated transformation. Twelve independent individuals (CD40R-1 ~ CD40R-12) were obtained. Nine of 12 individuals showed clear reductions in the expression level of *OsHsp18.0-CI* (Fig. 3b). At the seedling stage, all individuals and WT were inoculated with RS105. The nine positive individuals exhibited significantly increased lesion length, with lesion lengths ranging from 2.36 cm to 3.32 cm, compared with average of 2.28 cm for WT plants (Fig. 3a). Consistent with these results, three negative individuals exhibited an average lesion length of 2.20 cm, similar to that in WT plants. In addition, the lesion length in the transgenic plants was significantly correlated (*r* = 0.933, α = 0.05, *n =* 12) with the expression level of *OsHsp18.0-CI.* Two individuals (CD40R-9 and CD40R-12) were chosen for further analysis at T_1_ generation. All positive T_1_ plants exhibited significantly increased lesion lengths compared with WT or negative plants segregated from the T_0_ transgenic lines (Fig. 3c and d). In the T_2_ generation, the progenies of CD40R-9 and CD40R-12 were more susceptible to RS105 than WT, and the bacteria populations in leaves of CD40R-12 was more than WT at 4 dpi and 8 dpi (Fig. 4a, b and c).

In addition, the transgenic plants were inoculation with several Chinese *Xoc* isolates including JLB2-24、HNB8-47、HGA1 and HGA2 to investigate the broad spectrum resistance of *OsHsp18.0-CI*. Compared with WT, the OE lines (CD39R-7) significantly enhanced resistance to all four Xoc isolates, and the RNAi lines (CD40R-12) were more susceptible (Fig. 4d). All the results strongly suggest that *OsHsp18.0-CI* positively regulates the resistance to *Xoc* in rice.

### Identification of Differentially Expressed Genes in Shendao 806 (CD39R-7) and Shendao 806 by RNA-seq

To identify genes involved in *OsHsp18.0-CI*-mediated resistance, RNA-seq of both infected (24 hpi) and non-infected leaves of CD39R-7(*OsHsp18.0-CI* over-expression line, OE) and Shendao 806 (WT) was conducted. An average of 24 million clean reads were obtained that mapped on the rice genome at an average rate of 87%, representing an average of 28,872 genes that were expressed in each sample (Additional file 1: Table S1).

**Fig. 5:**
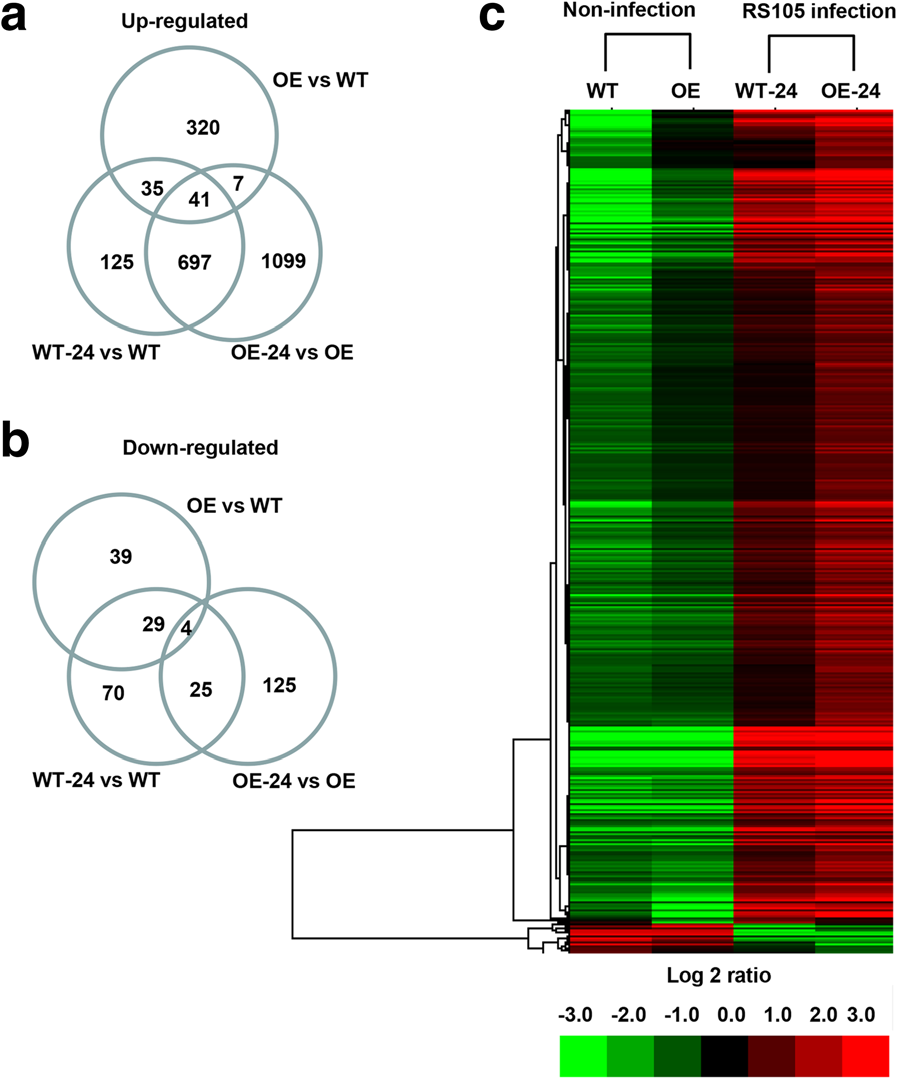
Gene expression profiling by RNA-seq. **a** Statistics for up-regulated genes among OE vs WT, WT-24 vs WT, and OE-24 vs OE. **b** Statistics for down-regulated genes. **c** Hierarchical clustering of differentially expressed genes. Total RNA was purified from non-infected and infected leaves (24 h post inoculation) of Shengdao 806 (WT) and CD39R-7 (OE). RNA-seq was performed on BGISEQ-500 by Beijing Genomic Institution. Differentially expressed genes were defined according to the combination of the absolute value of log2-Ratio ≥1 and FDR ≤ 0.001

Because the introduced gene could directly trigger the plant defense response regardless of the inoculation of pathogens, we first analyzed the differentially expressed genes (DEGs) under non-infected conditions. Compared with non-infected WT (WT), a total of 403 up-regulated genes and 72 down-regulated genes were detected in non-infected CD39R (OE, Fig. 5a and b, Additional file 2: Table S2). GO functional annotation of the DEGs indicated that the up- and down-regulated genes could be classified into 28 categories, as shown in Additional file 3: Figure S1, such as response to endogenous stimulus, signal transduction, cellular process, response to abiotic stimulus, response to stress, and response to biotic stimulus.

To further compare the DEGs activated by inoculation of RS105 between WT and OE, the transcriptional profiles were also analyzed between infected WT (WT-24) and WT and between infected OE (OE-24) and OE. Compared with WT, a total of 898 up-regulated and 128 down-regulated genes were identified in WT-24 from the RNA-seq data, and 1844 up-regulated and 154 down-regulated genes were identified in OE-24 compared with non-infected OE. All DEGs were classified into 40 different functional categories according to Gene Ontology analysis using Plant MetGenMap (http://bioinfo.bti.cornell.edu/cgi-bin/MetGenMAP/home.cgi; ﻿Joung et al. 2009), as described in Additional file 4: Figure S2. In both OE-24 and WT-24, most of the DEGs belonged to the function categories of response to endogenous stimulus, cellular process, response to stress, signal transduction, response to abiotic stimulus and response to biotic stimulus (Additional file 4: Figure S2). The distributions of the DEGs in the different function categories were also similar (*r* = 0.993, α = 0.05, *n =* 40). More DEGs were identified in OE-24 than in WT-24. In particular, almost all of the functional categories were enriched in more DEGs from OE-24 than WT-24. This result implies that a similar but enhanced biological process is involved in *OsHsp18.0-CI*-mediated resistance to *Xoc* as in the basal defense of WT against *Xoc*.

Of the 898 and 1844 up-regulated genes in WT and OE induced by RS105, a total of 738 genes were common between OE and WT (Fig. 5a). GO analysis of all 738 genes indicated that the distributions of the DEGs in 34 different function categories were similar to the results described in Additional file 5: Figure S3A. Similarly, out of 128 and 154 down-regulated genes in WT and OE induced by RS105, 29 genes in 12 function categories were commonly repressed in both OE and WT (Fig. 5b, Additional file 5: Figure S3B). Of the 738 commonly up-regulated genes, RS105 induced higher expression of 694 genes in OE-24 than in WT-24 (Fig. 5c). Among the 29 commonly down-regulated genes, 16 genes were repressed more strongly by RS105 in OE-24 than in WT-24 (Fig. 5c). In general, most of the common DEGs exhibited greater changes at the transcriptional level in the transgenic plants than in WT post inoculation with RS105. This result is also consistent with an enhanced version of resistance to *Xoc* in *OsHsp18.0-CI*-overexpressing plants.

### Differential Expression of Pathogenesis-related (*PR*) Genes and Genes Related to the Biosynthesis of Salicylic Acid and Jasmonic Acid

**Fig. 6:**
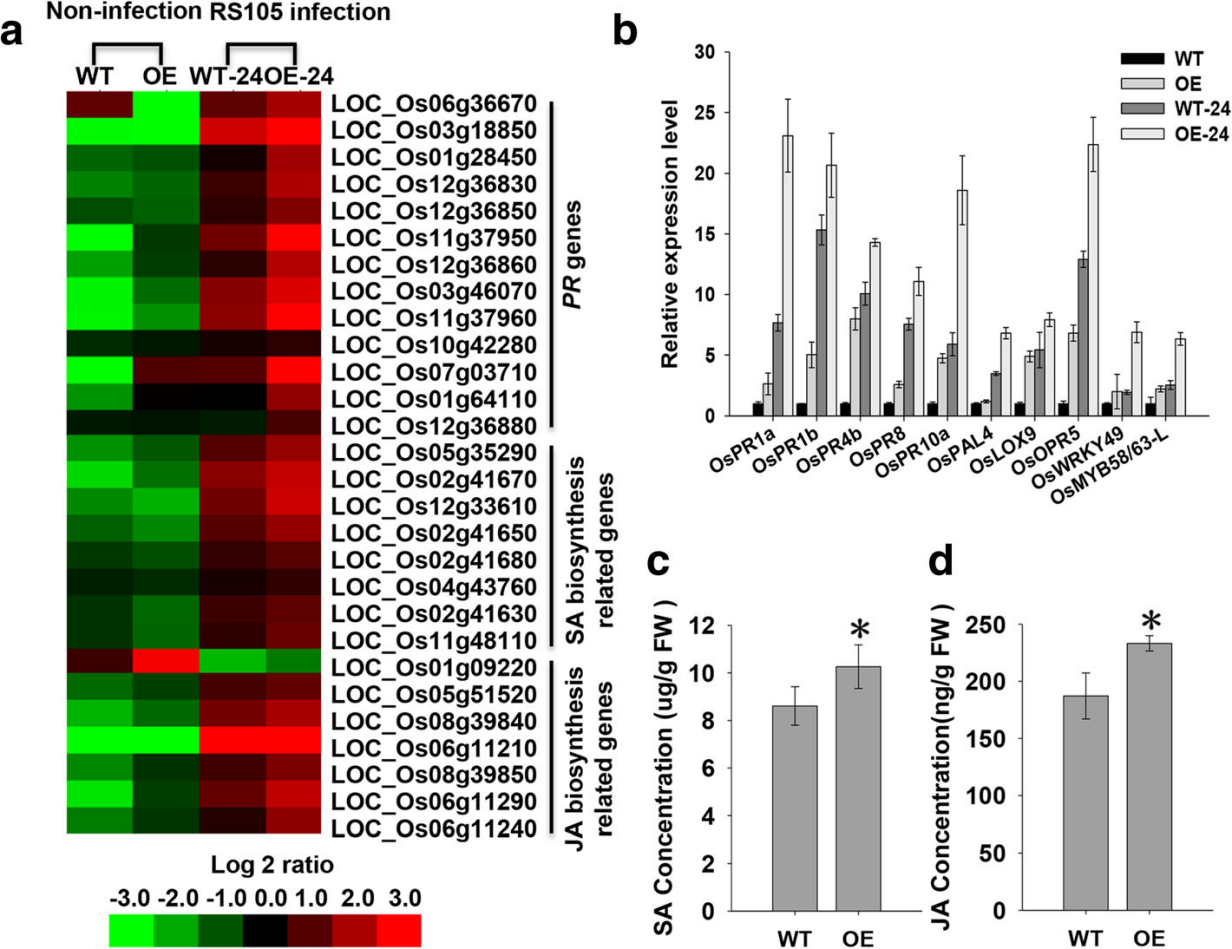
Expression of pathogenesis-related genes and SA and JA biosynthesis-related genes in wild-type and transgenic lines. **a** Hierarchical clustering analysis of *PR* genes and SA and JA biosynthesis-related genes using RNA-seq data. **b** Quantitative RT-PCR analysis of 10 selected *PR* genes to validate the RNA-seq data of up-regulated genes. **c** The content of SA. **d** The content of JA. The data are shown as the means ± SD of three biological replicates. The experiments were repeated two times

Many previous reports indicated that the induced expression of pathogenesis-related (*PR*) genes and increased content of salicylic acid (SA) and jasmonic acid (JA) are important for enhanced resistance to *Xoc* in rice (Shen et al. 2010; Guo et al. 2012; Feng et al. 2016). Among 738 commonly up-regulated genes and 29 down-regulated genes, we identified a total of 28 genes, including 13 *PRs*, and 8 and 7 genes involved in the biosynthesis of SA and JA, respectively (Fig. 6a). All 13 *PR* genes exhibited higher induction of expression by RS105 in OE-24 than in WT-24 (Fig. 6a). The salicylate biosynthesis and jasmonic acid biosynthesis pathway were significantly changed with a *P* value less than 0.05. All 8 SA biosynthesis-related genes belong to a gene family that encodes putative phenylalanine ammonia-lyases (PALs) involved in SA synthesis. In addition, all of these genes were more highly expressed in OE-24 than in WT-24 after inoculation with RS105 (Fig. 6a). Of the seven DEGs identified as JA biosynthesis-related genes, six were more highly expressed in OE-24 than in WT-24 and encoded phospholipase A2 (LOC_Os05g51520), lipoxygenase (*OsLOX8* and *OsLOX9*), and 12-oxophytodienoate reductase (LOC_Os06g11290, LOC_Os06g11240 and LOC_Os06g11210) (Fig. 6a). Consist with the expression of SA- and JA-biosynthesis-related genes, the content of SA and JA has increased in OE line (CD39R-7) comparing to WT (Fig. 6c and d). In addition, we also identified several DEGs involved in the ethylene or auxin signaling pathway among the 738 commonly up-regulated genes, such as ethylene-responsive transcription factors (LOC_Os04g32620, LOC_Os11g0677, LOC_Os07g47790, LOC_Os04g46400, LOC_Os02g32140, LOC_Os03g08470, LOC_Os01g64790), *OsIAA2* (LOC_Os01g09450) and *OsPILS1* (LOC_Os09g31478) (Additional file 6: Table S3).

In addition to the profiling analysis, the expression levels of ten DEGs were validated by quantitative reverse transcriptase-PCR. The results confirmed the RNA-seq data with regard to the expression patterns of all selected genes (Fig. 6b). In general, compared with WT, all ten genes were more highly expressed in OE post inoculation with RS105 (Fig. 6b).

### Overexpression of *OsHsp18.0-CI* Results in Small Fitness Costs

Lots of defense-related genes are resulted in enhancing resistance to particular pathogens as well as causing costly plant fitness, such as dwarfism, accelerated senescence, sterility, or reduced seed production (Chen et al. 2014; Liu et al. 2015). To estimate the resistance costs modulating by *OsHSP18.0-CI*, we compared several agronomic traits with wild type Shengdao 806, CD39R-7 (OE) and CD40R-12 (RNAi). Compared to Shengdao 806, the *OsHsp18.0-CI* OE lines exhibited a small decreased phenotype in plant height, 100-grain weight, grain width and grain thickness, but not significant differences in the tillers numbers per plant, grain number per panicle and grain length (Additional file 7: Figure S5). However, the RNAi lines showed no significant differences compared with Shengdao 806 for all tested traits (Additional file 7: Figure S5). Collectively, these results show that the overexpression of *OsHsp18.0-CI* also has a slight negatively impact on rice fitness.

## Discussion

### OsHsp18.0-CI Plays Important Roles in Abiotic and Biotic Stresses

HSPs are molecular chaperones that specifically prevent irreversible protein aggregation (Waters 2013). As a large family, HSPs have an important role in thermo tolerance. The expression of HSPs is regulated by heat shock transcription factors (HSFs), which bind to heat shock elements or promoters (Kumar et al. 2009; Scharf et al. 2012). Roles of HSPs in biotic stresses have also been identified, such as HSP90 and HSP70 (Park and Seo 2015). sHSPs are the most abundant of HSPs in plants and against environmental stresses such as heavy metals, drought, cold and oxidative stress (Scharf et al. 2012). Some types of biotic stresses also activate the expression of plant sHSPs (Sarkar et al. 2009; Waters 2013; Li et al. 2015). With the exception of the increased susceptibility of *N. benthamiana* to *Ralstonia solanacearum* induced by silencing of *NtHSP17* (Maimbo et al. 2007), few reports have indicated that plant sHSPs can protect against plant pathogens.

OsHsp18.0-CI is one of 23 sHSP members in rice (Sarkar et al. 2009). OsHsp18.0-CI also belongs to class I sHSPs in the database sHSPdb (Jaspard and Hunault 2016). OsHsp18.0-CI has been reported to function in tolerance to salt and cadmium stresses and interact with the N-terminus of viral RdRp (Cui et al. 2013; Ham et al. 2013; Li et al. 2015). In this study, we found that the overexpression of *OsHsp18.0-CI* enhanced resistance against bacterial pathogenic *Xoc* in rice (Fig. 2 and Fig. 4). These results provide additional evidence that OsHsp18.0-CI plays important roles in both abiotic and biotic stresses.

### OsHsp18.0-CI Positively Regulates Resistance to *Xoc* in Rice

Induction of the expression of *OsHsp18.0-CI* by the fungus *M. grisea* and at least seven rice viruses has previously been reported (Sarkar et al. 2009; Li et al. 2015). In the present study, the expression of *OsHsp18.0-CI* was activated by inoculation with a strain of *Xoc*, a bacterial pathogen (Fig. 1). This result indicates that *OsHsp18.0-CI* belongs to the *DR* genes, which respond to a broad spectrum of pathogens in rice. Consistent with the up-regulation of expression by *Xoc*, overexpression of *OsHsp18.0-CI* increased resistance, whereas repression of the expression of *OsHsp18.0-CI* enhanced susceptibility to *Xoc*. The expression level of *Xoc*-responsive related genes reported previously were also analyzed in the OE transgenic plants, the negatively regulators such as *DEPG1*, *NRRB*, *OsWRKY45-1* and *OsMPK6* have a lower expression level in both non-infected and infected OE lines compared with WT, and the positive regulated gene *GH3-2* has a higher level in OE lines than WT (Additional file 8: Figure S4). These results imply that *OsHsp18.0-CI* positively regulates resistance to *Xoc* in rice. Consistent with the positive regulation mechanism, the content of SA and JA (Fig. 6c), the expression of PR genes (Fig. 6a), and the resistance costs (Additional file 7: Figure S5) were slightly higher in *OsHsp18.0-CI* overexpressing plants than in wild-type.

The chromosomal location of *OsHsp18.0-CI* is in the region of 8834823 to 8835802 on chromosome 3 (http://rice.plantbiology.msu.edu). This location coincides with the location of mapped *qBlsr*3a, a minor QTL for bacterial leaf streak resistance in rice Acc8558 (Tang et al. 2000). Recently, many *DR* genes that co-localize with QTLs have been identified that may explain the small effect of these QTLs (Kou and Wang 2010; Feng et al. 2016). Taken together, *OsHsp18.0-CI* may help explain the effect of *qBlsr*3a.

### OsHsp18.0-CI Mediates a Basal Defense that Differs from that of *Rxo1*-mediated Resistance

Plant have developed two types of pathogen perception. On the external face of the host cell, plants use extracellular pattern recognition receptors (PRRs) to recognize conserved microbial molecules known as pathogen-associated molecular patterns (PAMPs), which induce PAMP-triggered immunity (PTI) or basal defense (Zipfel 2014). The second class of perception involves the recognition of cognate effectors by resistance (R) proteins that induce effector-triggered immunity (ETI; Cui et al. 2015). Both PTI and ETI induce immune responses and consequently modulate the expression of genes in the host, referred to as differentially expressed genes (DEGs) or defense-responsive (*DR*) genes (Kou and Wang 2010).

To explore the possible resistance mechanism mediated by *OsHsp18.0-CI*, we compared the differential expression profiles between *OsHsp18.0-CI* in our study and *Rxo1*, the only cloned qualitative resistance gene to bacterial leaf streak, which was introduced into japonica rice variety 9804 (Zhao et al. 2005; Zhou et al. 2010). Zhou et al. (2010) determined differential expression profiles using microarray between 9804-*Rxo1* and 9804 in response to inoculation with *Xoc* strain FJR5. In total, 1239 and 963 up-regulated genes were screened out in 9804-Rxo1 and 9804 induced by *Xoc*. Of those up-regulated genes, only 143 genes were common between the 9804-Rxo1 and 9804 (Zhou et al. 2010). In the present study, of the 1844 and 898 up-regulated genes in *OsHsp18.0-CI* OE and WT induced by RS105, a total of 738 genes were common between OE and WT (Fig. 5a). Because susceptible wild type is normally considered to only harbor basal defense after inoculation with *Xoc*, whereas the resistant variety harbors a qualitative resistance gene, the higher ratio of commonly up-regulated genes with wild-type in *OsHsp18.0-CI* OE plants suggests that the *OsHsp18.0-CI*-mediated resistance activates a basal defense similar to that found in wild-type, in contrast to the *Rxo1*-mediated resistance.

Consistent with this difference from to *Rxo1*-mediated resistance, the distribution of up-regulated genes in different function categories was similar in response to inoculation with *Xoc* strain RS105 in *OsHsp18.0-CI* OE plants and wild-type (Additional file 4: Figure S2) but were quite different between 9804-Rxo1 and 9804 (Zhou et al. 2010). As further evidence, 120 and 79 differentially regulated transcription factor (TF) genes were identified in the 9804-Rxo1 and 9804 plants infected by *Xoc* strain FJR5, respectively. Among them, only 7 TFs were commonly identified in both 9804-Rxo1 and 9804 (Zhou et al. 2010; Additional file 9: Table S4). In this study, 119 and 71 TF genes were differentially expressed in *OsHsp18.0-CI OE* and WT, and a total of 50 TFs were commonly identified in both *OsHsp18.0-CI OE* and WT (Additional file 9: Table S4). Among the 119 and 120 differentially expressed TFs in *OsHsp18.0-CI OE* and 9804*-Rxo1*, 104 and 105 TFs were specifically up-regulated or repressed in *OsHsp18.0-CI OE* and 9804*-Rxo1*, respectively. Fifteen TFs were common DEGs in *OsHsp18.0-CI OE* and 9804*-Rxo1*. Among them, only 7 TFs exhibited the same expression pattern, and 8 exhibited opposite expression patterns in *OsHsp18.0-CI OE* and 9804*-Rxo1.* For example, OsWRKY45, a negative regulator of BLS resistance (Tao et al. 2009), exhibited down-regulated expression in *OsHsp18.0-CI* OE but up-regulated expression in 9804*-Rxo1*.

Several *DR* genes have been implicated in BLS resistance by inducing the expression of *PR* genes and increasing the content of SA and JA (Tao et al. 2009; Shen et al. 2010; Guo et al. 2014; Feng et al. 2016). After inoculation with RS105, we detected significantly elevated expression of many genes, including SA and JA biosynthesis-related genes and 10 *PR* genes (Fig. 6). Interestingly, the expression of these genes was also elevated in WT after inoculation with RS105, but the expression levels were lower than in *OsHsp18.0-CI* OE*.* Together with the above evidence, these results indicate that *OsHsp18.0-CI-*mediated resistance occurs in a basal defense manner.

## Conclusions

Our study revealed that *OsHsp18.0-CI* positively regulates resistance to bacterial leaf streak in rice. Resistance mediated by the overexpression of *OsHsp18.0-CI* may prime an enhanced version of basal defense after inoculation with *Xoc*.

## Methods

### Plant Materials and Growth Condition

A japonica BLS-susceptible rice variety, Shengdao 806, which is widely planted in Shandong Province of China, was used in this study. Seeds were kindly provided by Dr. Fangying Yao of Shandong Academy of Agricultural Science (Jinan, Shandong, China). All rice plants were grown in greenhouse at a temperature of 28 ± 2 °C, relative humidity of 85-100%, and photoperiod of 16 h, as described previously (Feng et al. 2016).

### Vector Construction and Rice Transformation

Genomic DNA was isolated from the leaves of Shengdao 806 with a Plant DNA Extraction Kit (CWBio, Beijing, China). Because there are no introns in *Os03g16030* (*OsHsp18.0-CI*), a 657-bp DNA containing the coding sequence of *OsHsp18.0-CI* was amplified by PCR using the specific primers OsHsp18.0-CI-1 F and OsHsp18.0-CI-1R (Additional file 10: Table S5). The PCR product was digested with *Bam*H I and *Kpn* I (New England Biolabs, MA, USA) and cloned into the binary vector pU1301 cleaved with *Bam*H I and *Kpn* I to prepare the construct for overexpressing *OsHsp18.0-CI* (pU1301-*OsHsp18.0-CI*) as described previously (Li et al. 2013).

To construct a RNAi vector suppressing the expression of *OsHsp18.0-CI*, the expected 480-bp DNA fragment containing a portion of the encoding region and the 3′-UTR was obtained by PCR using the primers OsHsp18.0-CI-2 F and OsHsp18.0-CI-2R (Additional file 10: Table S5). The appropriate restriction sites were introduced into the PCR product for subsequent cloning steps (*Spe* I and *Kpn* I at the 5′ end and *Sac* I and *Bam*H I at the 3′ end). The PCR product was first cloned into *Kpn* I-/*Bam*H I-digested ds1301vector and then into *Spe* I-/*Sac* I-digested ds1301 to obtain ds1301-*OsHsp18.0-CI* as described previously (Li et al. 2013). The PCR product was inserted approximately 1.1 kb from the intron of the rice alcohol dehydrogenase (*Adh*) gene.

The constructs of pU1301-*OsHsp18.0-CI* (OE) and ds1301-*OsHsp18.0-CI* (RNAi) were introduced into Shengdao 806 using the standard *Agrobacterium*-mediated transformation system described previously (Li et al. 2013). Transgenic plants were selected with hygromycin for two constructs in our studies. OE individuals were further validated by PCR with the forward primer UbiF (Additional file 10: Table S5) derived from the sequence of the ubiquitin promoter and the reverse primer *OsDRXoc8*-1R (Additional file 10: Table S5). For RNAi individuals, positive plants were identified using a pair of specific primers, ds1301-F and ds1301-R, which were described previously (Feng et al. 2016).

### Pathogen Inoculation and Disease Assessment

The virulent *Xoc* strain RS105, JLB2-24, HNB8-47, HGA1 and HGA2 were grown on polypeptone-sucrose-agar medium (10 g l^−1^ polypeptone, 1 g l^−1^ glutamic acid, 10 g l^−1^ sucrose and 15 g l^−1^ agar) at 28 °C for 2 days and then resuspended in sterile 10 mM MgCl_2_ to OD_600_ = 0.5. More than five newly expanded leaves were infiltrated at three positions with a non-needle syringe at the seedling stage (Liu et al. 2014). The lesion length was scored at 14 days post-inoculation (dpi). The population count experiments were progressed as previously described (Triplett et al. 2016), rice leaves of OE, RNAi and WT plants harvested at three specific time points (0, 4, 8 dpi).

### RNA Extraction and Real-time RT-PCR

Total RNA was isolated from 100 mg of rice leaves using TRI Reagent (Sigma Aldrich, USA) following the procedures described in the manual. First-strand cDNA synthesis was performed using a HiFiScript gDNA Removal cDNA Synthesis Kit (CWBIO, Beijing, China) according to the recommended protocols. Quantitative real-time PCR was performed on a QuantStudio™ 6 Flex Real-Time System (Applied Biosystems, USA) with UltraSYBR Mixture (CWBIO, Beijing, China).

The following PCR program was used: 95 °C for 30 s, followed by 40 cycles of 95 °C for 5 s, 55 °C for 20 s, and 72 °C for 30 s. A heat dissociation curve (55-95 °C) was checked after the final PCR cycle to determine the specificity of the PCR amplification. The gene expression levels relative to the rice *OsActin* (LOC_Os03g50890) gene were analyzed using the 2^-⊿⊿Ct^ analysis method. Genes expression levels were analyzed by qRT-PCR assays, which were repeated at least twice with triplicate runs. The primer sequences for each detected gene are listed in Additional file 10: Table S5. The primers qRT-1 F and qRT-1R were used to check the expression levels of *OsHsp18.0-CI* in the O*sHsp18.0-CI*-OE lines, and for the *OsHsp18.0-CI* -RNAi plants, the primers qRT-2 F and qRT-2R were used.

### RNA-seq and Analysis

RNA samples were collected from non-infected and infected leaves of Shengdao 806 and Shengdao 806 (CD39R-7), respectively. Library construction and sequencing were performed on a BGISEQ-500 by Beijing Genomic Institution (www.genomics.org.cn, BGI, Shenzhen, China). Clean-tags were mapped to the reference genome and genes available at the Rice Genome Annotation Project (http://rice.plantbiology.msu.edu) with a perfect match or one mismatch. The original sequence data have been submitted to the database of the NCBI Sequence Read Archive (http://trace.ncbi.nlm.nih.gov/Traces/sra) under the accession number SRP079496. For gene expression analysis, the matched reads were calculated and then normalized to RPKM using RESM software (Li and Dewey 2011). The significance of the differential expression of genes was defined by the bioinformatics service of BGI according to the combination of the absolute value of log2-Ratio ≥1 and FDR ≤ 0.001. KOG functional classification, Gene Ontology (GO) and pathway annotation and enrichment analyses were based on the NCBI COG (https://www.ncbi.nlm.nih.gov/COG/), Gene Ontology Database (http://www.geneontology.org/) and KEGG pathway database (http://www.genome.jp/kegg/), respectively. The software Cluster and Java Treeview were used for hierarchical cluster analysis of gene expression patterns (de Hoon et al. 2004; Saldanha 2004).

### Quantification of Phytohormone

The leaves of four-week-old seedlings were used for phytohormone quantification. Each of 100 ug samples were prepared and quantified using the HPLC-MS/MS system as reported by Xu et al. (2016). Three biological replicates were analyzed.

### Statistical Analysis

As described previously (Feng et al. 2016), standard deviations were checked visually by error bars, and statistical significance was determined by analysis of variance. The data were subjected to one-way analysis of variance, and the mean differences were compared by paired *t* test. *P* values <0.05 were considered significant. Correlation analysis was performed using SPSS software.

## Additional files

Additional file 1: Table S1. Summary of the sequence assembly after RNA-seq. (DOCX 13 kb)

Additional file 2: Table S2 List of DEGs in OE under non-infected conditions. (XLSX 29 kb)

Additional file 3: Figure S1. GO functional annotation of DEGs between OE and WT under non-infected conditions. (TIF 1894 kb)

Additional file 4: Figure S2. The numbers of DEGs in different function categories in OE and WT under infection with RS105. (TIF 2022 kb)

Additional file 5: Figure S3. The numbers of DEGs in different function categories for 738 commonly up-regulated genes (A) and 29 commonly down-regulated genes (B) between WT and OE induced by RS105. (TIF 2568 kb)

Additional file 6: Table S3. List of DEGs involved in the ethylene or auxin signaling pathway in OE and WT under infection with RS105. (DOCX 14 kb)

Additional file 7: Figure S5. Effects of *OsHsp18.0-CI* in transgenic plants. (A) Comparison of transgenic lines and wild type Shengdao 806 at the tillering stage, bar = 10 cm. (B-D) Grain morphology of transgenic lines and Shengdao 806, bar = 10 mm. (E) Plant height of transgenic lines and Shengdao 806 at the tillering stage, mean values were calculated from measurement on at least 10 individuals. (F) Number of tillers per plant in the wild-type and transgenic plants, Data were obtained from at least 10 individuals. (G) Number of grains per panicle in the wild-type and transgenic plants, mean values were calculated from measurement on at least 20 individuals. (G-K) Phenotype statistics of seeds from WT and transgenic plants, mean values were calculated from measurement on at least 20 individuals. The bars represent the means ± SD. “**” indicate extremely significant differences between wild-type and transgenic plants at *P* = 0.01 by Student’s *t* test. (TIF 5760 kb)

Additional file 8: Figure S4. The expression level of *Xoc*- responsive related genes. (TIF 356 kb)

Additional file 9: Table S4. List of the TF genes differentially regulated by *Xoc* in 9804, 9804-*Rxo1*, WT and *OsHsp18.0-CI*-OE. (XLSX 29 kb)

Additional file 10: Table S5. Primers used in this study. (DOCX 15 kb)

## Abbreviations

BLS: Bacterial leaf streak
DEG: Differentially expressed gene
dpi: Days post inoculation
DR: Defense-related
hpi: Hours post inoculation
HSPs: Heat shock proteins
OE: Overexpression
PR: Pathogenesis related
WT: Wild-type
*Xoc*: *Xanthomons oryzae* pv. *oryzicola*
