# Supplementary material for: Overexpression of OsHSP18.0-CI Enhances Resistance to Bacterial Leaf Streak in Rice
Source: Rice (N Y). 2017 Apr 17;10:12. doi: 10.1186/s12284-017-0153-6 (PMC5393982; doi:10.1186/s12284-017-0153-6)
Supplement: Supplementary file 1 — Summary of the sequence assembly after RNA-seq. (DOCX 13 kb) [file 12284_2017_153_MOESM1_ESM.docx]

Table S1. Summary of the sequence assembly after RNA-seq

| Sample | Raw Data Size (bp) | Raw Reads Number | Clean Data Size (bp) | Clean Reads Number | Total Mapped Reads (%) | Unique Match(%) | Number of expressed genes |
| --- | --- | --- | --- | --- | --- | --- | --- |
| WT | 1,206,861,900 | 24,137,238 | 1,206,636,750 | 24,132,735 | 87.55 | 80.32 | 28,037 |
| OE | 1,206,839,800 | 24,136,796 | 1,206,614,350 | 24,132,287 | 87.00 | 79.59 | 28,771 |
| WT-24 | 1,206,846,900 | 24,136,938 | 1,206,567,200 | 24,131,344 | 86.85 | 79.97 | 28,978 |
| OE-24 | 1,206,841,800 | 24,136,836 | 1,206,272,600 | 24,125,452 | 86.86 | 79.46 | 29,701 |
