# Supplementary figures and images for: Overexpression of OsHSP18.0-CI Enhances Resistance to Bacterial Leaf Streak in Rice
Source: Rice (N Y). 2017 Apr 17;10:12. doi: 10.1186/s12284-017-0153-6 (PMC5393982; doi:10.1186/s12284-017-0153-6)

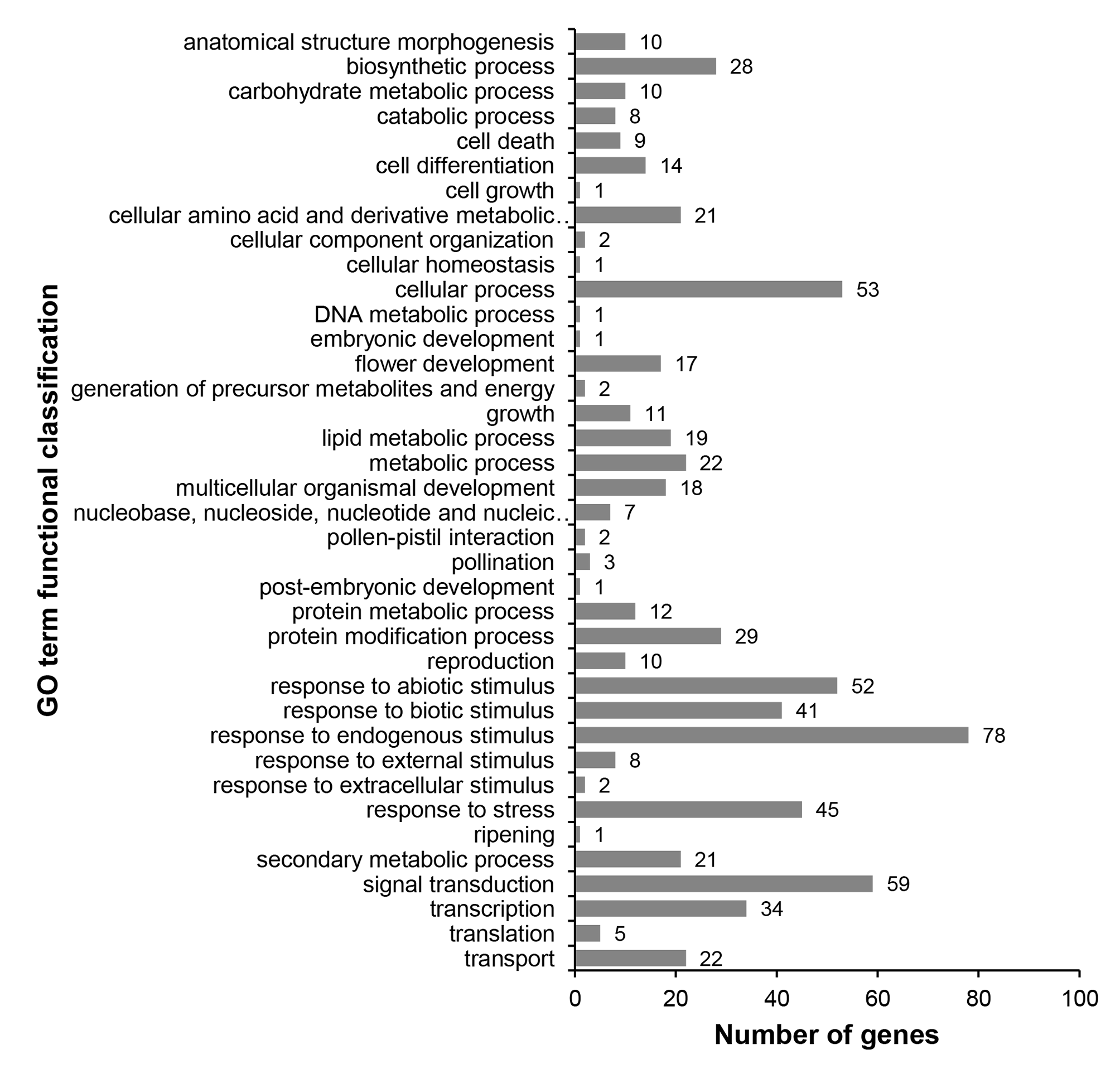

Supplement: Supplementary file 3 — GO functional annotation of DEGs between OE and WT under non-infected conditions. (TIF 1894 kb) [file 12284_2017_153_MOESM3_ESM.tif]

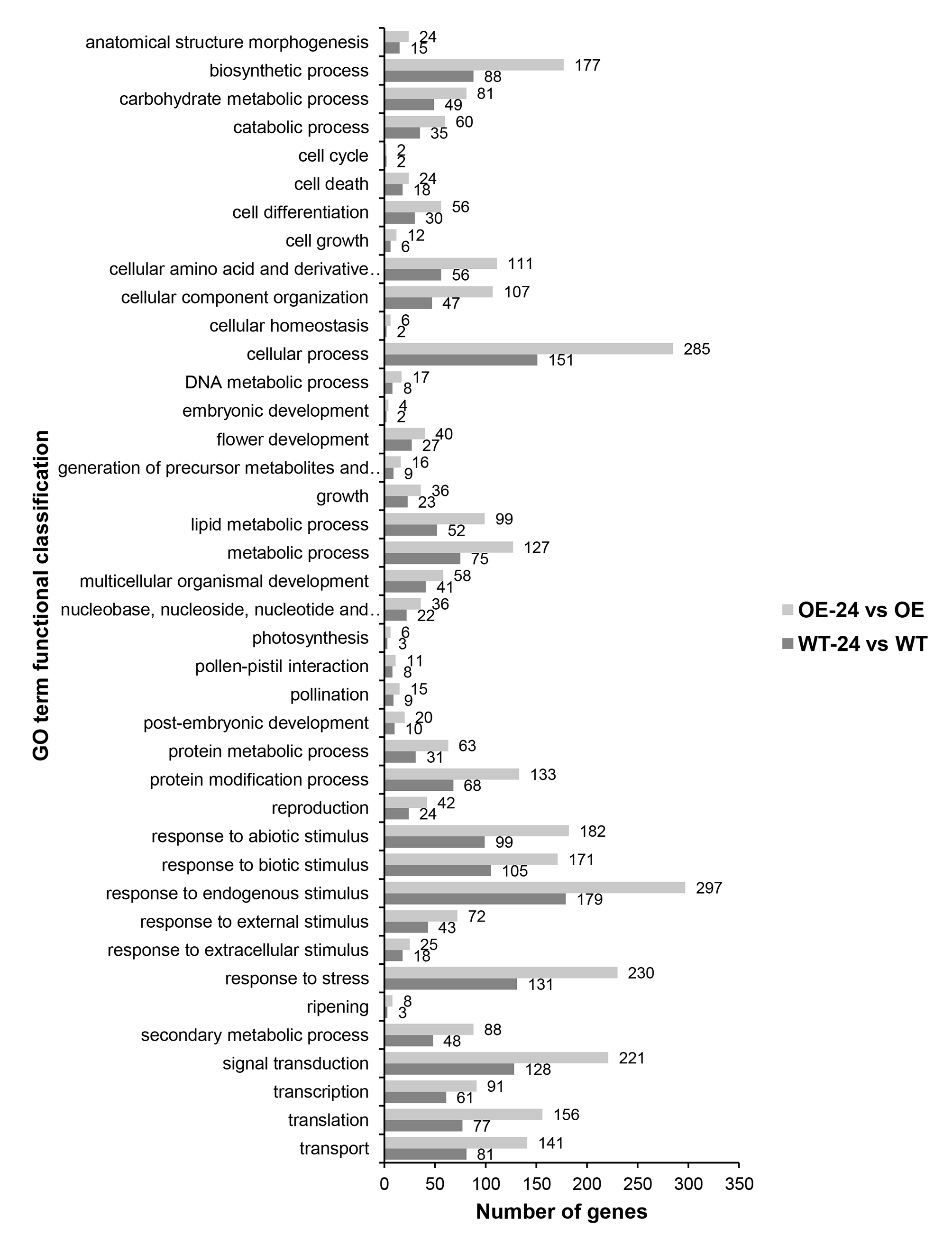

Supplement: Supplementary file 4 — The numbers of DEGs in different function categories in OE and WT under infection with RS105. (TIF 2022 kb) [file 12284_2017_153_MOESM4_ESM.tif]

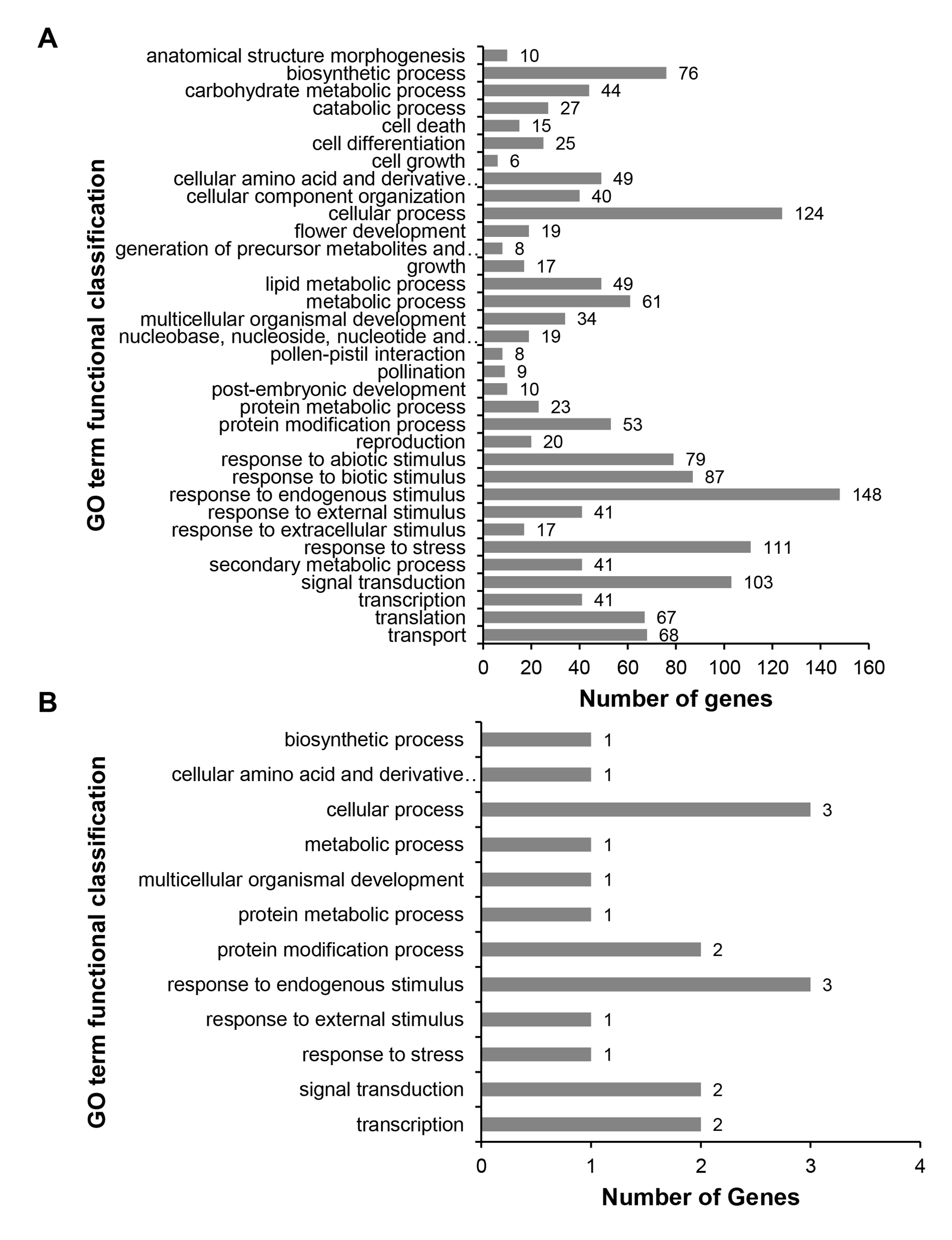

Supplement: Supplementary file 5 — The numbers of DEGs in different function categories for 738 commonly up-regulated genes (A) and 29 commonly down-regulated genes (B) between WT and OE induced by RS105. (TIF 2568 kb) [file 12284_2017_153_MOESM5_ESM.tif]

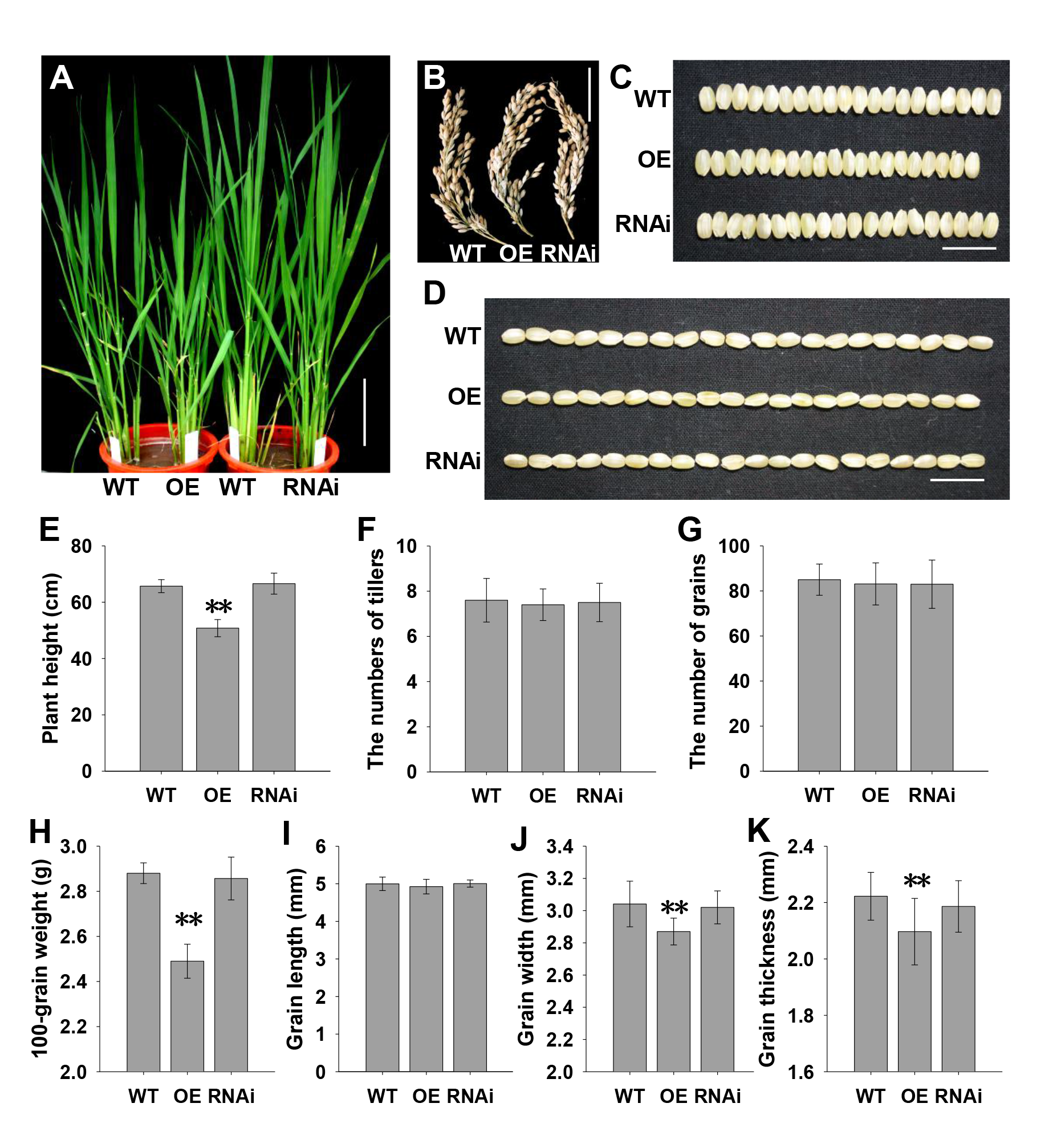

Supplement: Supplementary file 7 — Effects of OsHsp18.0-CI in transgenic plants. (A) Comparison of transgenic lines and wild type Shengdao 806 at the tillering stage, bar = 10 cm. (B-D) Grain morphology of transgenic lines and Shengdao 806, bar = 10 mm. (E) Plant height of transgenic lines and Shengdao 806 at the tillering stage, mean values were calculated from measurement on at least 10 individuals. (F) Number of tillers per plant in the wild-type and transgenic plants, Data were obtained from at least 10 individuals. (G) Number of grains per panicle in the wild-type and transgenic plants, mean values were calculated from measurement on at least 20 individuals. (G-K) Phenotype statistics of seeds from WT and transgenic plants, mean values were calculated from measurement on at least 20 individuals. The bars represent the means ± SD. “**” indicate extremely significant differences between wild-type and transgenic plants at P = 0.01 by Student’s t test. (TIF 5760 kb) [file 12284_2017_153_MOESM7_ESM.tif]

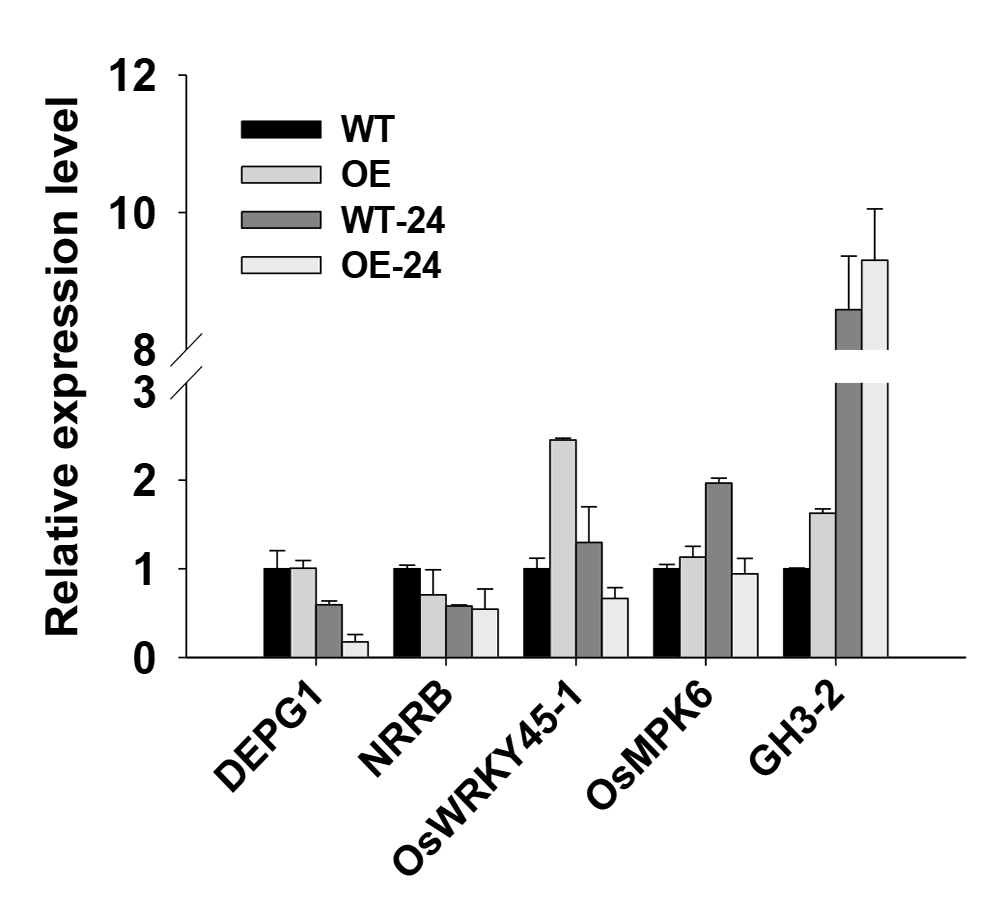

Supplement: Supplementary file 8 — The expression level of Xoc- responsive related genes. (TIF 356 kb) [file 12284_2017_153_MOESM8_ESM.tif]
