# Supplementary material for: Overexpression of OsHSP18.0-CI Enhances Resistance to Bacterial Leaf Streak in Rice
Source: Rice (N Y). 2017 Apr 17;10:12. doi: 10.1186/s12284-017-0153-6 (PMC5393982; doi:10.1186/s12284-017-0153-6)
Supplement: Supplementary file 6 — List of DEGs involved in the ethylene or auxin signaling pathway in OE and WT under infection with RS105. (DOCX 14 kb) [file 12284_2017_153_MOESM6_ESM.docx]

**Additional file3: Table S3.** List of DEGs involved in the ethylene or auxin signaling pathway in OE and WT under infection with RS105.

| Gene ID | log2 Ratio | | Description |
| --- | --- | --- | --- |
|  | WT-24/WT | OE-24/OE |  |
| LOC_Os07g47790 | 5.30 | 7.95 | AP2 domain containing protein, expressed |
| LOC_Os04g46400 | 2.95 | 3.87 | AP2 domain containing protein, expressed |
| LOC_Os02g32140 | 5.86 | 3.64 | AP2 domain containing protein, expressed |
| LOC_Os03g08470 | 1.84 | 2.21 | AP2 domain containing protein, expressed |
| LOC_Os01g64790 | 1.60 | 2.00 | AP2 domain containing protein, expressed |
| LOC_Os11g06770 | 6.41 | 6.66 | ethylene-responsive transcription factor ERF110, putative, expressed |
| LOC_Os04g32620 | 3.02 | 1.81 | ethylene-responsive transcription factor ERF114, putative, expressed |
| LOC_Os01g09450 | 1.16 | 1.13 | OsIAA2 - Auxin-responsive Aux/IAA gene family member, expressed |
| LOC_Os09g31478 | 1.77 | 1.88 | auxin efflux carrier component, putative, expressed |
