# Supplementary material for: Overexpression of OsHSP18.0-CI Enhances Resistance to Bacterial Leaf Streak in Rice
Source: Rice (N Y). 2017 Apr 17;10:12. doi: 10.1186/s12284-017-0153-6 (PMC5393982; doi:10.1186/s12284-017-0153-6)
Supplement: Supplementary file 10 — Primers used in this study. (DOCX 15 kb) [file 12284_2017_153_MOESM10_ESM.docx]

**Additional file5: Table S5 primers used in this study**

| Gene ID | Primer name | Primer Sequence(5′-3′)^a^ |
| --- | --- | --- |
| LOC_Os03g16030 | OsHsp18.0-CI-1F | ATGGGTACCTGAGAATTGAGATCACCCTCTT |
|  | OsHsp18.0-CI-1R | CGGGATCCGGACCAGATTTGACGCTTT |
| LOC_Os03g16030 | OsHsp18.0-CI-2F | AAGACTAGTGGTACCGCATCTTCCCGTCCTTCC |
|  | OsHsp18.0-CI-2R | AAGGAGCTCGGATCCGGACCAGATTTGACGCTTT |
| LOC_Os03g50890 | OsActin1F | TGCTATGTACGTCGCCATCCAG |
|  | OsActin1R | AATGAGTAACCACGCTCCGTCA |
|  | UbiF | TTTTAGCCCTGCCTTCATACGC |
|  | ds1301-F | TTCTAATCCCCAATCCAAA |
|  | ds1301-R | TAGGCGTCTCGCATATCTC |
| LOC_Os03g16030 | qRT-1F | GGTGGAGAGCTTCGATTCGA |
|  | qRT-1R | GGACCAGATTTGACGCTTTTATTT |
| LOC_Os03g16030 | qRT-2F | CAACCAAAAAACAGCAAGACACA |
|  | qRT-2R | CCCAGAGGTCGAGGGAGAAG |
| LOC_Os07g03710 | OsPR1a-F | CGTCTTCATCACCTGCAACTACTC |
|  | OsPR1a-R | CATGCATAAACACGTAGCATAGCA |
| LOC_Os01g28450 | OsPR1b-F | GGCAACTTCGTCGGACAGA |
|  | OsPR1b-R | CCGTGGACCTGTTTACATTTTCA |
| LOC_Os11g37960 | OsPR4b-F | GGCGGAAAACAATTGGGATT |
|  | OsPR4b-R | TCCATCCATACTTCTGGCGC |
| LOC_Os01g64110 | OsPR8-F | GAGACGAGCGACCTGGTGTC |
|  | OsPR8-R | CCCGTGAGCCCGTCATAGTA |
| LOC_Os12g36880 | OsPR10a-F | CCCTGCCGAATACGCCTAA |
|  | OsPR10a-R | CTCAAACGCCACGAGAATTTG |
| LOC_Os02g41680 | OsPAL4-F | AGCACATCTTGGAGGGAAGCT |
|  | OsPAL4-R | GCGCGGATAACCTCAATTTG |
| LOC_Os08g39840 | OsLOX9-F | GCATCCCCAACAGCACATC |
|  | OsLOX9-R | TTTGGGAGTGACATATTGG |
| LOC_Os06g11210 | OsOPR5-F | TTTTCTTGGCGAACCCTGACT |
|  | OsOPR5-R | TCCAACAATTGGGTCTTGAGTGT |
| LOC_Os05g49100 | OsWRKY49-F | TTCGCGTTCATGACCAAGAG |
|  | OsWRKY49-R | CTCCTGGGAAATGGGCTGTT |
| LOC_Os02g46780 | OsMYB58/63-L-F | ACTCGGGAACAAGTGGTCCA |
|  | OsMYB58/63-L-R | GCGACACCCTCTTCTTCAGG |
| LOC_Os01g55940 | GH3-2qRTF | GTGCGTGTGTAATTTCTCGTGTTT |
|  | GH3-2qRTR | GTAACTACCACTGGACAGCATGATCT |
| LOC_Os05g30220 | DEPG1-qRTF | GATTGGAGCTGTCACGGATT |
|  | DEPG1-qRT-R | TCGTTCATGTTCCTGAGCTG |
| LOC_Os10g40100 | NRRB-qRT-F | CTTATTTTGCTGCATGGGGT |
|  | NRRB-qRT-R | CTGTTTTGGACTTGGGCAAT |
| LOC_Os05g25770 | WRKY45-1qRT-F | TTCCTTGTTGATGTGTCGTCTCA |
|  | WRKY45-1qRT-R | CCCCCAGCTCATAATCAAGAAC |
| LOC_Os10g38950 | MPK6qRT-F | TTGCTACGAGGGCTAAAATATGTG |
|  | MPK6qRT-R | GGAACAAATTGCTTGGCTTCA |

^a^ Restriction site sequences are underlined
